# Supplementary material for: The Streptomyces viridochromogenes product template domain represents an evolutionary intermediate between dehydratase and aldol cyclase of type I polyketide synthases
Source: Commun Biol. 2022 May 26;5:508. doi: 10.1038/s42003-022-03477-8 (PMC9135731; doi:10.1038/s42003-022-03477-8)
Supplement: Supplementary file 2 — Supplementary Information [file 42003_2022_3477_MOESM2_ESM.pdf]

# Supplementary Information

**The *Streptomyces viridochromogenes* Product Template Domain represents An Evolutionary Intermediate between Dehydratase and Aldol Cyclase of Type I Polyketide Synthases**

Yuanyuan Feng,[1,†] Xu Yang,[1,†] Huining Ji,[1] Zixin Deng,[1] Shuangjun Lin,[1] Jianting Zheng[1,2✉]

[1] State Key Laboratory of Microbial Metabolism, School of Life Sciences and Biotechnology, Shanghai Jiao Tong University, Shanghai, China

[2] Joint International Research Laboratory of Metabolic & Developmental Sciences, Shanghai Jiao Tong University, Shanghai, China

[†] Y.F. and X.Y. contributed equally to this work

✉ E-mail: jtzheng@sjtu.edu.cn

Supplementary Information:

Supplementary Fig. 1 – Supplementary Fig. 16.

Supplementary Table 1 – Supplementary Table 2

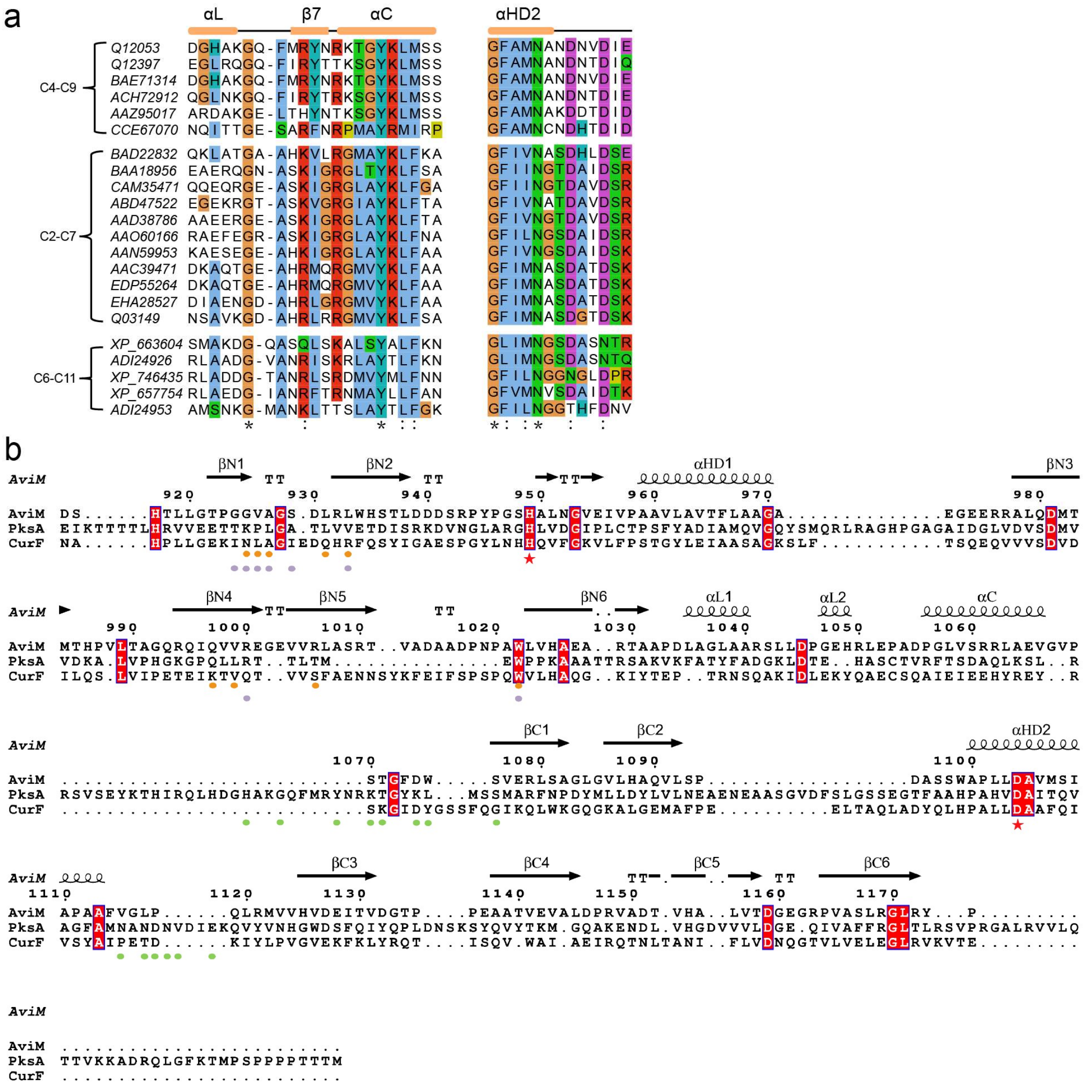


Supplementary Fig. 1. Multiple sequence alignment. a. PT-specific sequences contribute to dimerization of PT domain in fungal NR-PKSs. **b.** Multiple sequence alignment of AviM PT, PksA PT and CurF. The ‘His-Asp’ catalytic dyads of AviM PT, PksA PT, CurF DH are highlighted with red asterisk. The interface residues of AviM PT, PksA PT, CurF are labelled with orange circles, green circles, purple circles, respectively. AviM PT and CurF utilizes N-terminal β sheets structural elements to form dimer conformation.

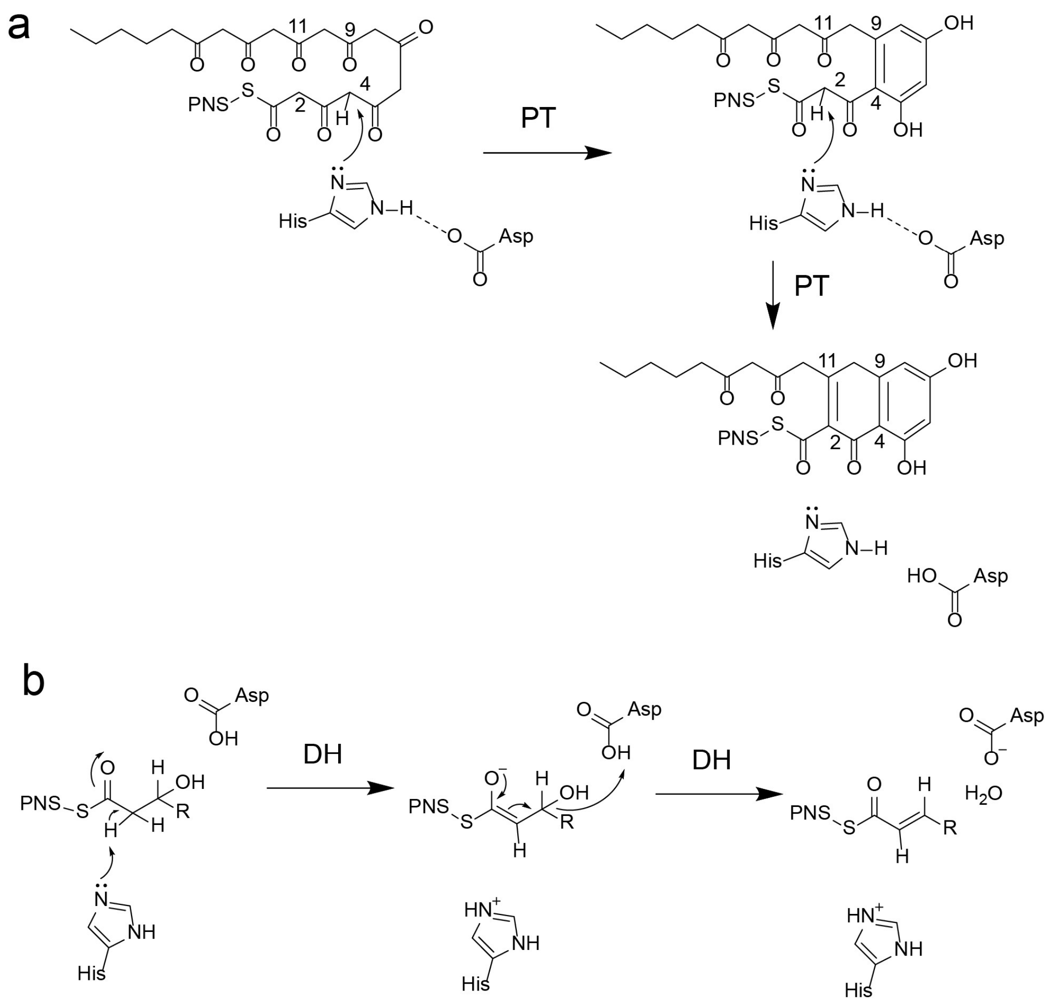


Supplementary Fig. 2. Catalytic mechanism of PT (a) and DH (b). a. The Asp residue form hydrogen bond with Nε of Histidine residue. The His acts as a catalytic base, leading to enolate formation at C4 to initiate first ring cyclization (C4–C9). The second ring is formed in the same manner as the first to achieve C2–C11 closure. **b.** A proton is abstracted from the α-position by a histidine residue, followed by elimination of water from the β-position by an aspartate residue.

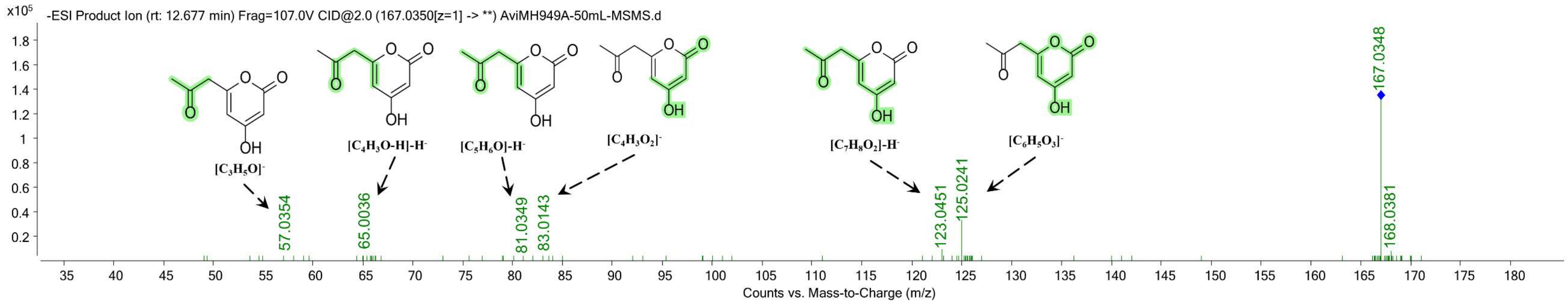


Supplementary Fig. 3. Mass spectrum. Collision-induced dissociation (CID) mass spectra of precursor ion scan (m/z 167.035, rt: 12.677 min. CE at 2 ev) by LC/ESI-MS. The potential fragments and formulars are labeled in the spectra

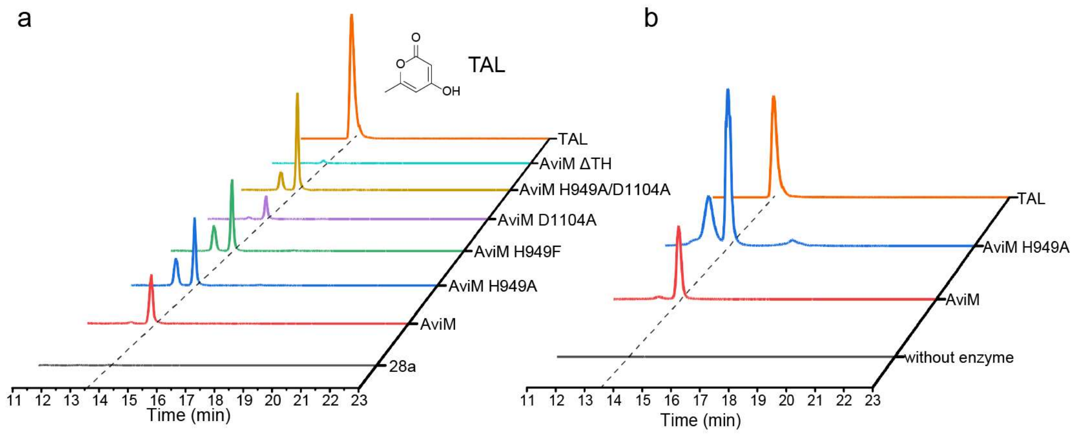


Supplementary Fig. 4. TAL was generated in the *in vivo* (a) and *in vitro* (b) assays. The traces shown are the selected ion monitoring (m/z: 125.0274) in the negative ionization mode of the LC-MS analysis.

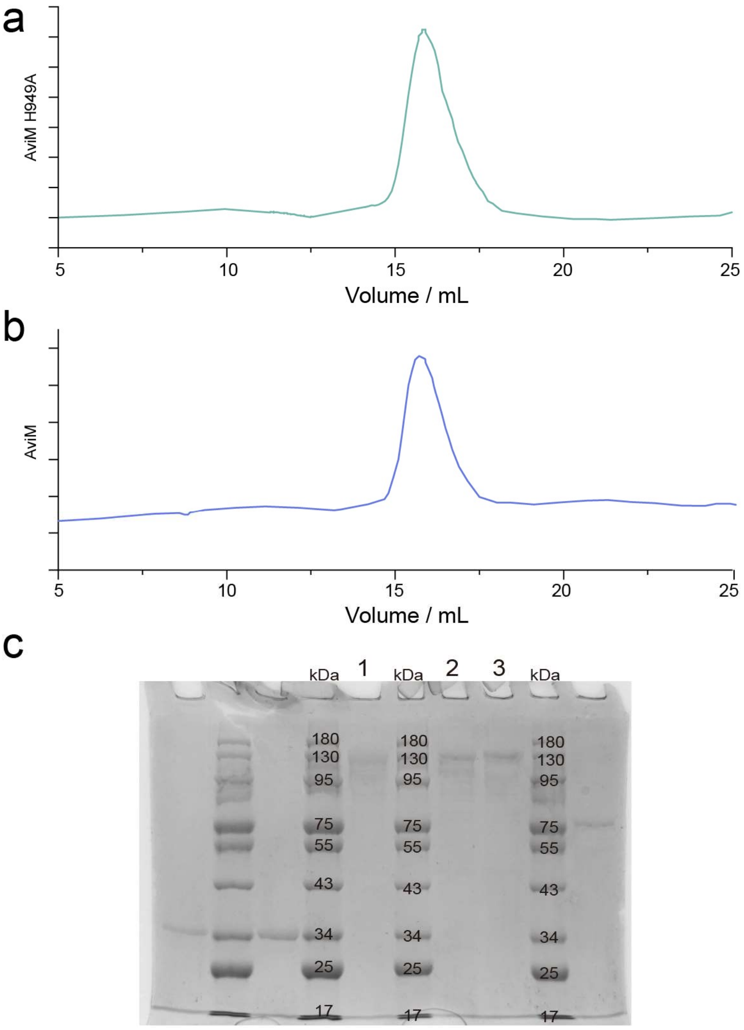


Supplementary Fig. 5. Size exclusion chromatography profiles and SDS-PAGE of AviM H949A and AviM. a. The SEC profile of AviM H949A. **b.** The SEC profile of AviM. **c.** Uncropped SDS-PAGE profile. lane 1 and 2 are AviM H949A; lane 3 are AviM. The lanes on the left are explained in Supplementary Fig. 8b.

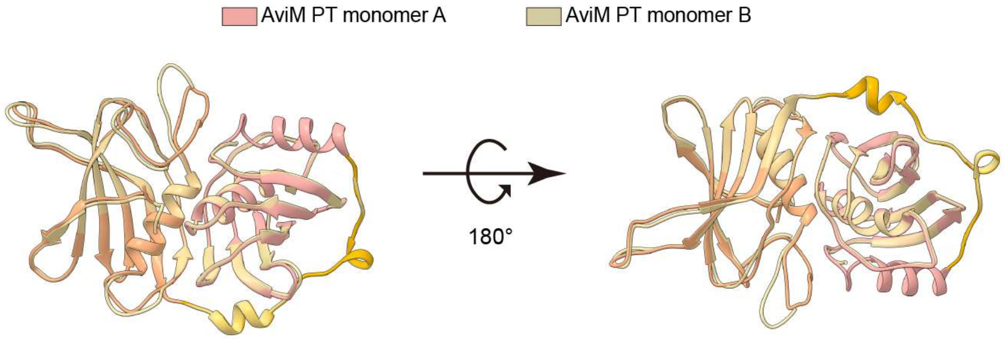


Supplementary Fig. 6. Two monomers of AviM PT were superposed and had essentially identical fold.

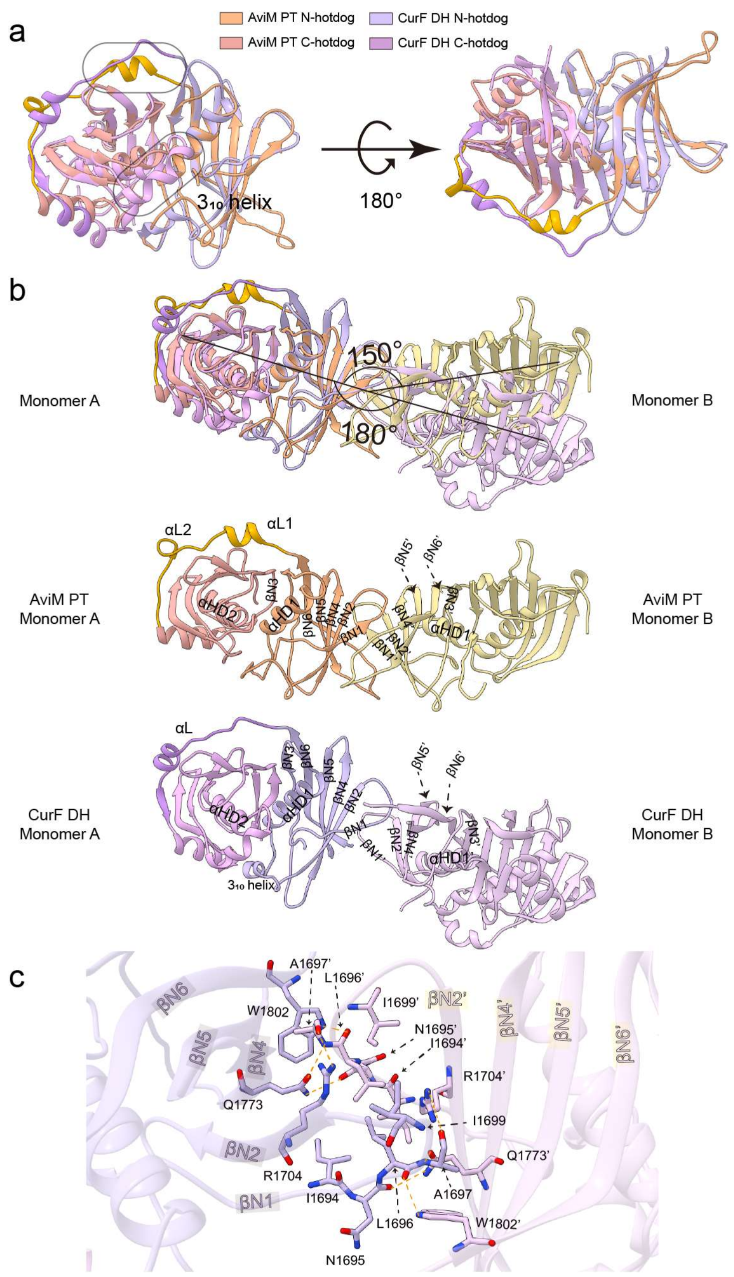


Supplementary Fig. 7. Structure differences between CurF DH (3KG6) and AviM PT. **a.** Comparison of CurF and AviM PT. CurF has an additional $3_{10}$ helix and lacks a linker helix. **b.** The monomer A of AviM PT and CurF are superposed. The angle formed by two monomers of AviM PT is ~150°. However, the angle formed by two monomers of CurF is ~180°. AviM PT and CurF both utilizes the β-hairpin turns between βN1 and βN2, and between βN5 and βN6 to form dimer interface. The monomer A and B of AviM PT are shown in orange and yellow, respectively. The monomer A and B of CurF DH are shown in dark purple and light purple, respectively. **c.** Dimer interface residues of CurF. The major protein interactions between two monomers of CurF are hydrogen bonds and hydrophobic interactions. Hydrogen bonds are indicated with orange dashed lines.

a

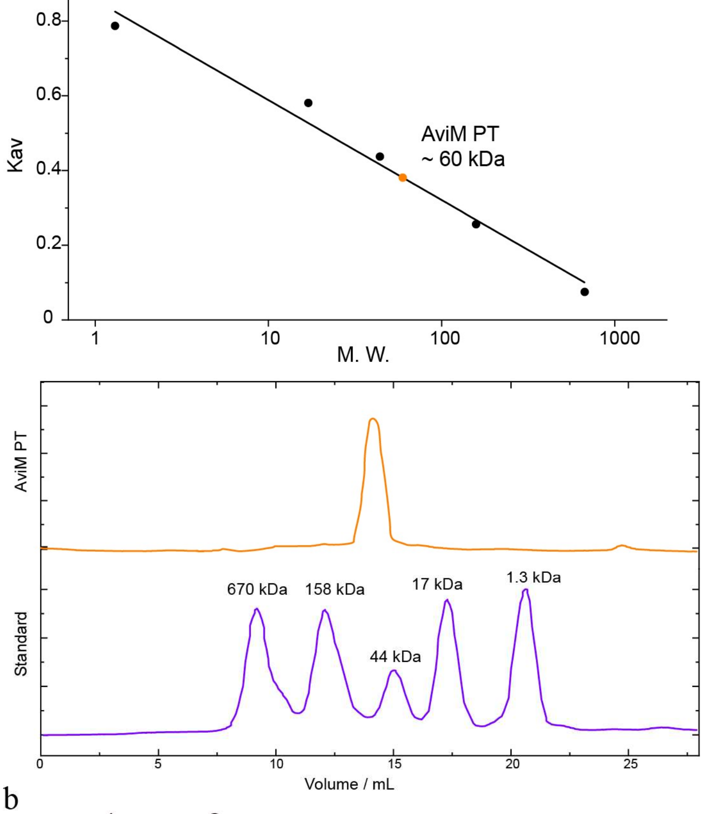


b

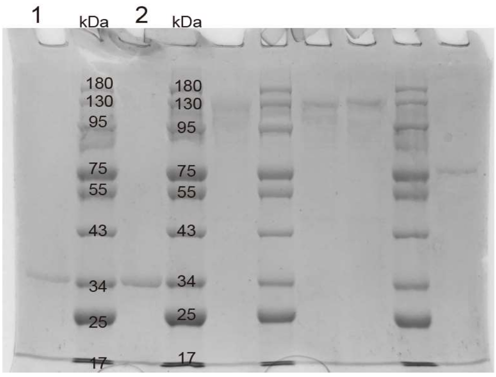


Supplementary Fig. 8. Molecular weight estimate by size-exclusion chromatography. a. AviM PT migrates at ~60 KDa (expected monomer mass: 29 kDa) compared to standards (FPLC buffer: 10 mM Tris, pH 7.0, 150 mM NaCl). b. The SDS-PAGE of AviM PT. b. The uncrpped SDS-PAGE of AviM PT (Lane1 and lane 2). The lanes on the right are explained in Supplementary Fig. 5c.

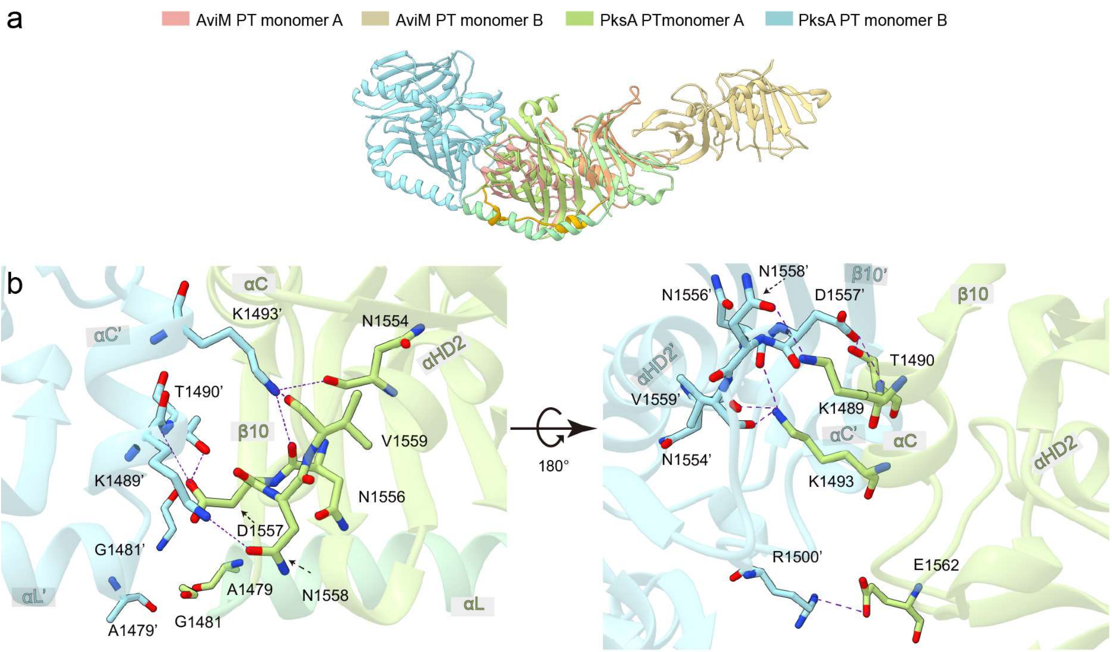


Supplementary Fig. 9. The dimer interfaces between AviM PT and PksA PT (PDB: 5KBZ). a. The monomer A of AviM PT and PksA PT were superposed. Ulike AviM PT, PksA PT utilizes C terminal structural elements to form dimer interface. The monomer A and B of AviM PT are shown in orange and yellow, respectively. The monomer A and B of PksA PT are shown in green and cyan, respectively. Hydrogen bonds are indicated with purple dashed lines. b. Residues on dimer interface of PksA PT.

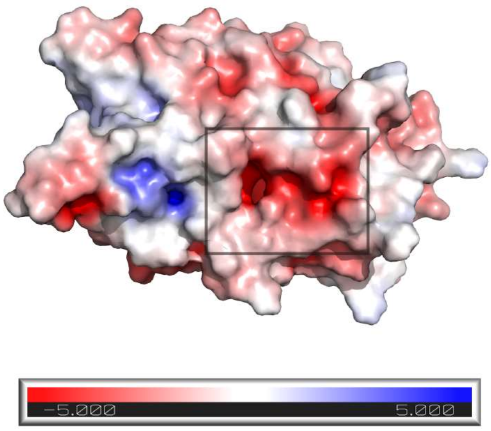


Supplementary Fig. 10. The Electrostatic potential surface of AviM PT. The dark square box indicated the negative substrate tunnel.

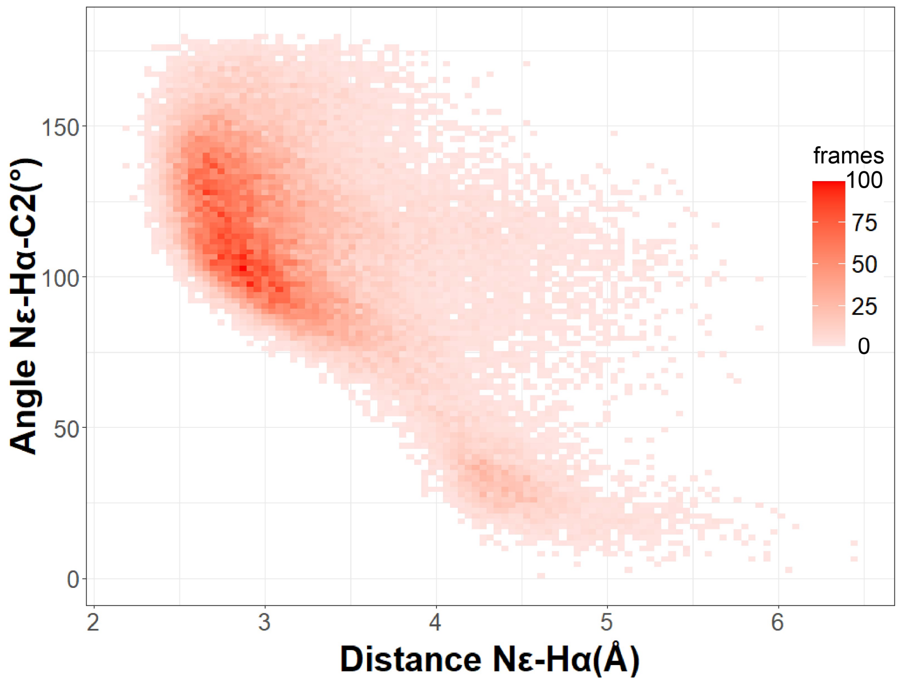


Supplementary Fig. 11. The distributions of d(Nε-Hα) and the angle (Nε-Hα-C2) in AviM PT-substrate system during MD simulations. The red color intensity indicates the distribution of frames. In most frames, the distance between the Nε atom of H949 and the Hα atom of the OSA was less than 3 Å, and the angle (Nε-Hα-C2) was greater than 110°, suggesting that the Nε of H949 could easily deprotonate the C2 of the substrate.

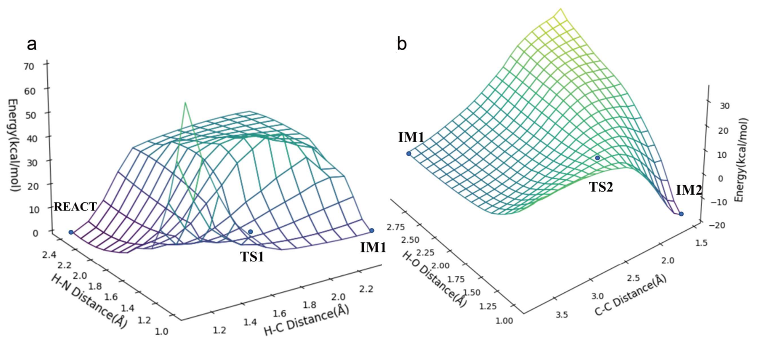


Supplementary Fig. 12. Two-dimensional potential energy surfaces (PESs) in the AviM PT-substrate system: a. the deprotonation of C2; b. the subsequent ring cyclization.

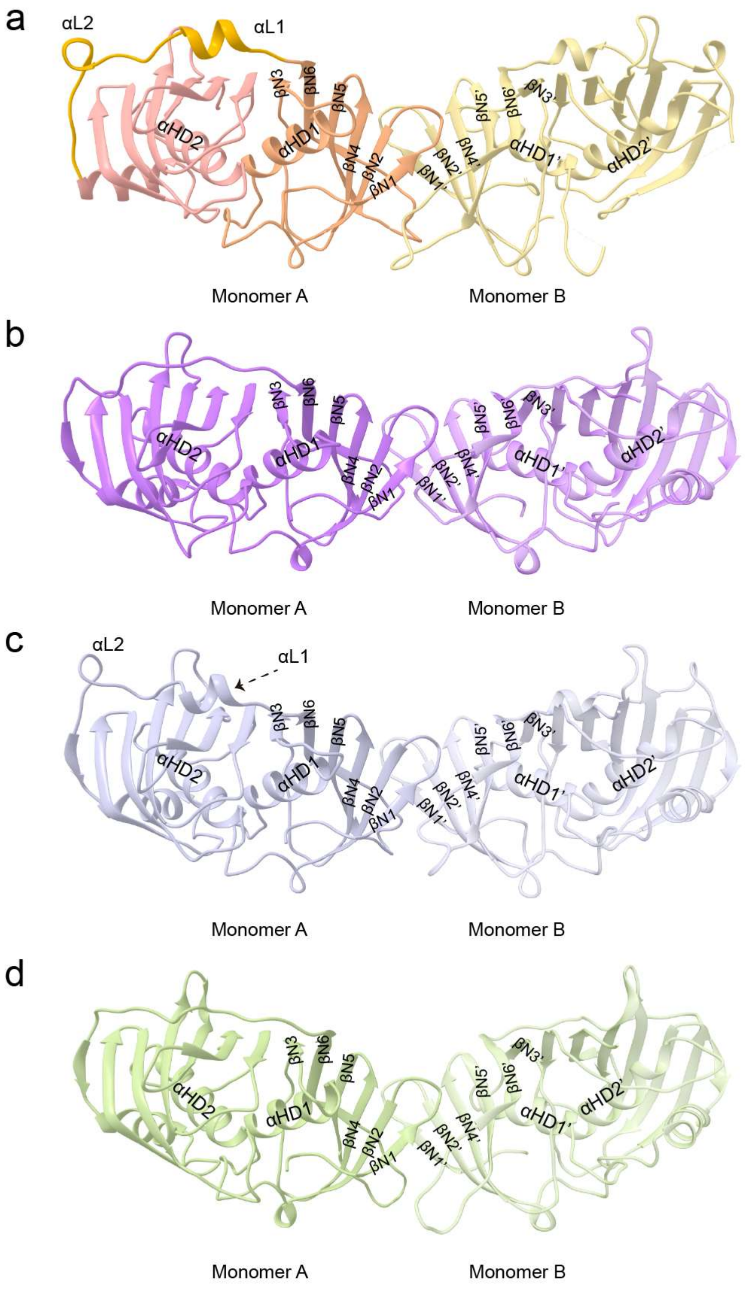


Supplementary Fig. 13. The overall structure of bacterial PTs, AviM (orange, a) ; CalO5 (Purple, b); L8PIV2 (gray, c); A0A1V0A0I3 (green, d). All bacterial PTs dimerize with N-terminal β sheets. All of the structures were constructed by Alphafold2.

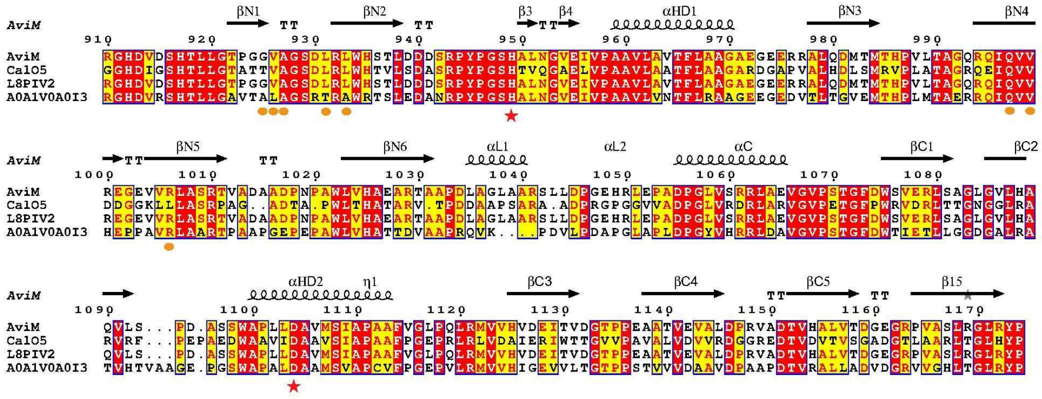

Supplementary Fig. 14. Multiple sequence alignment of bacterial PTs. The ‘His-Asp’ catalytic dyads of AviM PT are conserver in four PTs and are highlighted with red asterisk. The interface residues of AviM PT are labelled with orange circles, which are conserved in bacterial PTs.

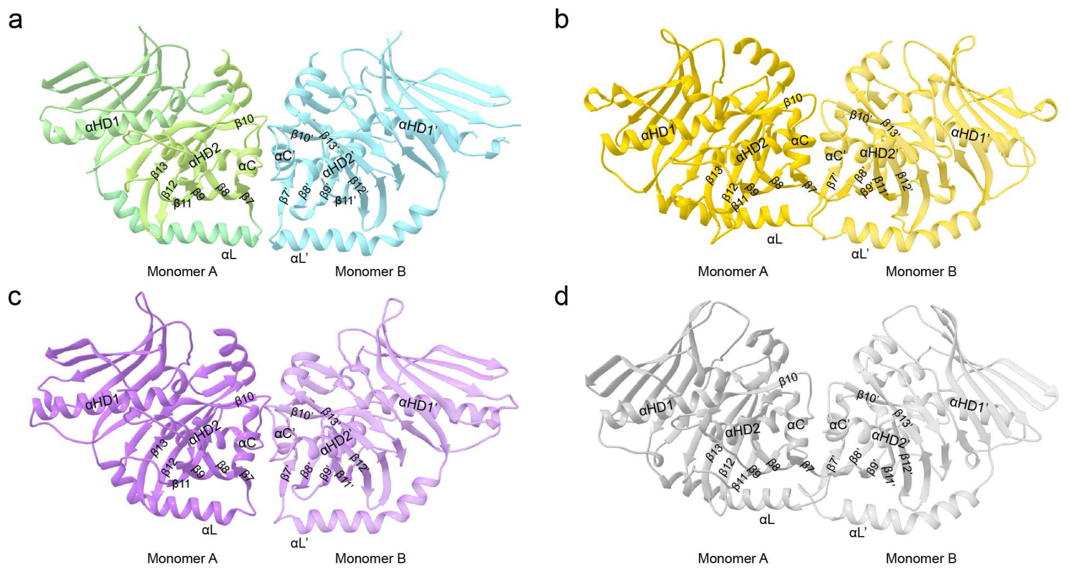


Supplementary Fig. 15. The overall structure of fungal PTs, PksA (green, a); AFL91703 (yellow, b); AAS92537 (purple, c); Q5AUX1 (gray, d). All fungal PTs dimerize through the structural elements of C-terminal hotdog, including the αC helix, the β7 strand, and the linker connecting αHD2 and β10. All of the structures were constructed by Alphafold2.

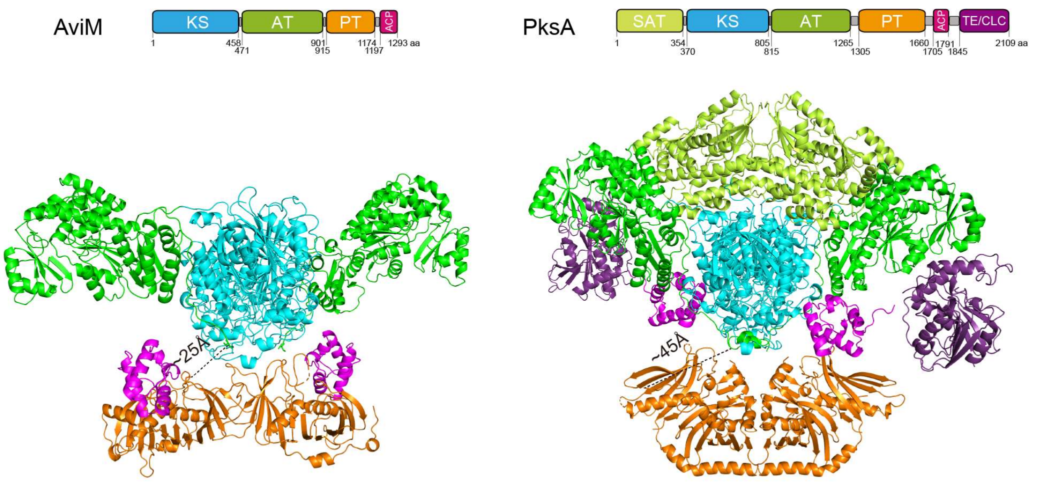


Supplementary Fig. 16. Cartoon representation of AviM and PksA model. The notable difference of PksA and AviM model is that the C-terminal of PksA AT is far away from the N-terminal of PksA PT and dimeric characteristic of PTs. The model of PksA SAT-KS-MAT, AviM KS-AT, and AviM ACP were built by AlphaFold2. PksA PT (PDB: 5KBZ), PksA ACP (PDB: 2KR5), and PksA TE/CLC (PDB: 3ILS) has been reported. SAT, limon; KS, blue; AT, green; PT, orange; ACP, magenta; TE/CLC, purple.

Supplementary Table 1. Primer list

| Primer | Sequence (5'→3') |
|---|---|
| AviMF | gcagatatacatatgaataccggcaacgacgaa |
| AviMR | gtggtgactagttcaggacgcatggccgtccgc |
| AviMPTF | gcagatatacatatggcgcgagcggccgagcgc |
| AviMPTR | tgattcgatgaattcacgggtagcgcaggccgcg |
| AviMD1104AF | gtcctgggccccgctgctggccgccgtgatgtcgatcgc |
| AviMD1104AR | ccagcagcggggcccaggacgaggcgtcgggcgagagc |
| AviMH949FF | cgcccgtacccgggcagcttcgccctcaacggcgtggaga |
| AviMH949FR | gctgcccgggtacgggcggctgtcgtcgtccagcgtgc |
| AviMH949AF | cgcccgtacccgggcagcgccgccctcaacggcgtggaga |
| AviMH949AR | gctgcccgggtacgggcggctgtcgtcgtccagcgtgc |
| AviMΔPTF | ccgagcgcggccacgacgtcgtggtcgagcagccggcc |
| AviMΔPTR | Gacgtcgtggccgcgctcggccgctcgcgccccggcga |

Supplementary Table 2. Protein sequences for phylogenetic analysis

| Protein | Strain | Accession code | Cyclization | Group |
|---|---|---|---|---|
| Bacterial DH | *Saccharopolyspora erythraea* | Q03132 | | |
| | *Lyngbya majuscula* | Q6DNE7 | | |
| | *Lyngbya majuscula* | Q6DNE2 | | |
| | *Lyngbya majuscula* | Q6DNE5 | | |
| | *Streptomyces albus subsp. albus* | H1ZZT4 | | |
| | *Streptomyces hygroscopicus* | Q54296 | | |
| | *Streptomyces albus subsp. albus* | H1ZZT7 | | |
| | *Streptomyces nodosus* | Q93NW6 | | |
| | *Streptomyces venezuelae* | Q9ZGI5 | | |
| | *Streptomyces nodosus* | Q93NX9 | | |
| | *Actinosynnema pretiosum subsp. auranticum* | Q8KUH3 | | |
| | *Actinosynnema pretiosum subsp. auranticum* | Q8KUH6 | | |
| | *Streptomyces natalensis* | Q9X993 | | |
| | *Streptomyces natalensis* | Q9EWA1 | | |
| | *Streptomyces natalensis* | Q9EWA3 | | |
| Bactrial PT | *Micromonospora echinospora* | Q8KND3 | C2-C7 | |
| | *Streptomyces viridochromogenes Tue57* | O05170 | C2-C7 | |
| | *Nonomuraea wenchangensis* | A0A1I0LQF6 | | |
| | *Nonomuraea solani* | A0A1H6F1V1 | | |
| | *Sphaerisporangium rubeum* | A0A7X0I997 | | |
| | *Nonomuraea rubra* | A0A7X0P230 | | |
| | *Nonomuraea wenchangensis* | A0A1I0ANF3 | | |
| | *Nonomuraea jabiensis* | A0A7W9LG52 | | |
| | *Nonomuraea solani* | A0A1H6F0G0 | | |
| | *Micromonospora sediminimaris* | A0A1I1T308 | | |
| | *Streptomyces viridochromogenes Tue57* | L8PIV2 | | |
| | *Nonomuraea sp. ATCC 55076* | A0A1V0A0I3 | | |
| Fungal PT | *Chaetomium chiversii* | ACM42403 | C2-C7 | I |
| | *Fusarium graminearum* | ABB90282 | C2-C7 | |
| | *Hypomyces subiculosus* | ACD39762 | C2-C7 | |
| | *Ceratocystis resinifera* | AAO60166 | C2-C7 | II |
| | *Colletotrichum Lagenaria* | BAA18956 | C2-C7 | |
| | *Ophiostoma piceae* | ABD47522 | C2-C7 | |
| | *Sordaria macrospora* | CAM35471 | C2-C7 | |
| | *Aspergillus fumigatus* A1163 | EDP55264 | C2-C7 | III |
| | *Aspergillus nidulans* FGSC A4 | Q03149 | C2-C7 | |
| | *Aspergillus niger* ATCC 1015 | EHA28527 | C2-C7 | |
| | *Aspergillus parasiticus* | Q12053 | C4-C9 | IV |
| | *Leptosphaeria maculans* | AAS92537 | C4-C9 | IV |
| | *Mycosphaerella pini* | AAZ95017 | C4-C9 | |
| | *Fusarium fujikuroi* | CCE67070 | C4-C9 | |
| | *Aspergillus ochraceoroseus* | ACH72912 | C4-C9 | |
| | *Aspergillus fumigatus* Af293 | XP_746435 | C6-C11 | V |
| | *Aspergillus nidulans* FGSC A4 | XP_657754 | C6-C11 | |
| | *Aspergillus nidulans* FGSC A4 | XP_663604 | C6-C11 | |

| | | | |
|---|---|---|---|
| *Aspergillus niger* CBS 513. 88 | XP_001394705 | C6-C11 | |
| *Aspergillus terreus* NIH2624 | XP_001217072 | C6-C11 | |
| *Aspergillus nidulans* FGSC A4 | XP_681652 | C2-C7 | |
| *Penicillium brevicompactum* | ADY00130 | C2-C7 | VI |
| *Aspergillus nidulans* FGSC A4 | XP_664052 | C2-C7 | |
| *Aspergillus nidulans* FGSC A4 | XP_658638 | C2-C7 | |
| *Aspergillus nidulans* FGSC A4 | XP_660990 | C2-C7 | |
| *Aspergillus nidulans* FGSC A4 | XP_658127 | C2-C7 | VII |
| *Aspergillus nidulans* FGSC A4 | XP_660834 | C2-C7 | |
