# Supplementary material for: The Streptomyces viridochromogenes product template domain represents an evolutionary intermediate between dehydratase and aldol cyclase of type I polyketide synthases
Source: Commun Biol. 2022 May 26;5:508. doi: 10.1038/s42003-022-03477-8 (PMC9135731; doi:10.1038/s42003-022-03477-8)
Supplement: Supplementary file 3 — Reporting Summary [file 42003_2022_3477_MOESM3_ESM.pdf]

nature portfolio

Corresponding author(s): Jianting Zheng

Last updated by author(s): 2022-04-25

Reporting Summary

Nature Portfolio wishes to improve the reproducibility of the work that we publish. This form provides structure for consistency and transparency in reporting. For further information on Nature Portfolio policies, see our Editorial Policies and the Editorial Policy Checklist.

Statistics

For all statistical analyses, confirm that the following items are present in the figure legend, table legend, main text, or Methods section.

n/a	Confirmed	
☐	☒	The exact sample size (*n*) for each experimental group/condition, given as a discrete number and unit of measurement
☐	☒	A statement on whether measurements were taken from distinct samples or whether the same sample was measured repeatedly
☒	☐	The statistical test(s) used AND whether they are one- or two-sided<br>*Only common tests should be described solely by name; describe more complex techniques in the Methods section.*
☒	☐	A description of all covariates tested
☒	☐	A description of any assumptions or corrections, such as tests of normality and adjustment for multiple comparisons
☒	☐	A full description of the statistical parameters including central tendency (e.g. means) or other basic estimates (e.g. regression coefficient) AND variation (e.g. standard deviation) or associated estimates of uncertainty (e.g. confidence intervals)
☒	☐	For null hypothesis testing, the test statistic (e.g. *F*, *t*, *r*) with confidence intervals, effect sizes, degrees of freedom and *P* value noted<br>*Give P values as exact values whenever suitable.*
☒	☐	For Bayesian analysis, information on the choice of priors and Markov chain Monte Carlo settings
☒	☐	For hierarchical and complex designs, identification of the appropriate level for tests and full reporting of outcomes
☒	☐	Estimates of effect sizes (e.g. Cohen's *d*, Pearson's *r*), indicating how they were calculated

Our web collection on statistics for biologists contains articles on many of the points above.

Software and code

Policy information about availability of computer code

Data collection	LC-MS data was collected using: Agilent 1290/6545 UHPLC-QTOF/MS, diffraction data of AviM PT was collected in BL18U1(SSRF) and indexed ,integrated, scaled using the HKL2000.
Data analysis	The phylogenetic analysis was done by using IQREE (v1.6.12). Computational analysis conducted with AutoDock software,Gaussian 16 program,AMBER software package (version 18). The MS/MS data was analyzed by MetFrag. The homology modeling of bacterial PTs and fungal PTs were constructed by Alphafold2. Experimental phases were determined using AutoSol in Phenix. The model was built in Coot and refined in Refmac.

For manuscripts utilizing custom algorithms or software that are central to the research but not yet described in published literature, software must be made available to editors and reviewers. We strongly encourage code deposition in a community repository (e.g. GitHub). See the Nature Portfolio guidelines for submitting code & software for further information.

Data

Policy information about availability of data

All manuscripts must include a data availability statement. This statement should provide the following information, where applicable:

- Accession codes, unique identifiers, or web links for publicly available datasets
- A description of any restrictions on data availability
- For clinical datasets or third party data, please ensure that the statement adheres to our policy

All data supporting the findings of this study are included in the paper and its supplementaru files. Structural factors and coordinates of AviM PT have been deposited in the Protein Data Bank under accession code 7VWK.

Field-specific reporting

Please select the one below that is the best fit for your research. If you are not sure, read the appropriate sections before making your selection.

☒ Life sciences ☐ Behavioural & social sciences ☐ Ecological, evolutionary & environmental sciences

For a reference copy of the document with all sections, see nature.com/documents/nr-reporting-summary-flat.pdf

Life sciences study design

All studies must disclose on these points even when the disclosure is negative.

Sample size	Sample size of at least three.
Data exclusions	No data was excluded.
Replication	Replicate experiments were successful.
Randomization	All strains for in vivo culture were randomly picked from LB with antibiotics agar plate.
Blinding	All in vivo and in vitro data were analyzed and checked by multiple authors and reviewed by the corresponding author.

Reporting for specific materials, systems and methods

We require information from authors about some types of materials, experimental systems and methods used in many studies. Here, indicate whether each material, system or method listed is relevant to your study. If you are not sure if a list item applies to your research, read the appropriate section before selecting a response.

Materials & experimental systems

n/a	Involved in the study	
☒	☐	Antibodies
☒	☐	Eukaryotic cell lines
☒	☐	Palaeontology and archaeology
☒	☐	Animals and other organisms
☒	☐	Human research participants
☒	☐	Clinical data
☒	☐	Dual use research of concern

Methods

n/a	Involved in the study	
☒	☐	ChIP-seq
☒	☐	Flow cytometry
☒	☐	MRI-based neuroimaging
